# Innovative strategies in kidney paired donation: single-center experience achieving the highest annual transplant volume globally

**DOI:** 10.3389/fimmu.2026.1623684

**Published:** 2026-03-03

**Authors:** Khalid A. AlMeshari, Dieter C. Broering, Dalia A. Obeid, Ali N. AlAli, Amal N. Algharabli, Noreen L. Pana, Tariq Z. Ali

**Affiliations:** 1 Department of Kidney and Pancreas Transplantation, King Faisal Specialist Hospital and Research Centre, Riyadh, Saudi Arabia; 2 Transplant Research and Innovation Department, Organ Transplant Centre of Excellence, King Faisal Specialist Hospital and Research Centre, Riyadh, Saudi Arabia

**Keywords:** ABO-incompatible transplant, HLA matching, HLA-incompatible transplant, kidney paired donation, kidney transplantation

## Abstract

**Background:**

Kidney Paired Donation (KPD) programs expand transplant opportunities for immunologically incompatible donor-recipient pairs. This study describes the operational framework and clinical outcomes of a high-volume, single-center KPD program, which became the highest-volume center globally in 2024.

**Methods:**

We analyzed all kidney transplants performed through our KPD program between January and December 2024. The program aimed to achieve full HLA and ABO compatibility for incompatible pairs, while also incorporating additional strategies: inclusion of compatible pairs to improve HLA matching, acceptance of ABO quasi-compatible matches (e.g., A2 donors to O or B recipients), low-risk HLA-incompatible matching for HLA-incompatible candidates with cPRA >80%, and ABO-incompatible matching for those with cPRA >95%.

**Results:**

A total of 135 patients (121 adults, 14 pediatrics) underwent KPD-facilitated transplantation, including 69 HLA-incompatible (51.1%), 37 ABO-incompatible (27.4%), and 29 compatible (21.5%) pairs. Females comprised 60.7% of the cohort, with a significantly higher proportion in the HLA-incompatible group (p < 0.001). HLA-incompatible recipients were older than others (mean age 42.5 years, p < 0.001). Most transplants (93.3%) occurred through 2- to 5-way closed chains, with the remainder via domino chains (6.7%). At baseline, 25% of patients were very highly sensitized (cPRA ≥95%) HLA- incompatible recipients, and ABO-incompatible recipients were blood group O individuals whose intended donors had A1 or B blood groups (high risk combinations). Following matching, 70% of patients achieved full HLA and ABO compatibility, while 30% underwent transplantation with acceptable immunologic risk (i.e. low-risk HLA incompatibility and/or ABO incompatibility). Early post-transplant outcomes were favorable, with a mean serum creatinine of 87.2 µmol/L. Acute rejection occurred in 6.7% of patients, antibody-mediated rejection in 0.7%, and graft loss in 0.7%.

**Conclusion:**

Our single-center experience demonstrates the feasibility and effectiveness of a high-volume KPD program in overcoming immunologic barriers to kidney transplantation. Strategic inclusion of compatible pairs, ABO quasi-compatible matching, low-risk HLA-incompatible, and ABO-incompatible matchings significantly increased access for difficult-to-match recipients. This model may serve as a replicable framework for other high-capacity transplant centers seeking to expand transplant access and improve outcomes for complex patient populations.

## 1 Introduction

End-stage renal disease (ESRD) affects millions worldwide, posing a growing public health challenge due to increasing incidence, rising healthcare costs, and its profound impact on quality of life. Kidney transplantation is recognized as the gold standard treatment for ESRD, offering superior patient survival, lower morbidity, and better health-related quality of life compared to long-term dialysis (1, 2). Despite these benefits, a persistent gap exists between the number of patients needing kidney transplants and the availability of suitable donors (3). Living donor kidney transplantation provides an opportunity to bridge this gap, but immunologic barriers often preclude direct transplantation.

The most significant immunologic challenges include ABO blood group incompatibility and the presence of donor-specific anti-HLA antibodies (DSAs), particularly in highly sensitized patients. Sensitization may arise from previous transplants, blood transfusions, or pregnancies and can drastically reduce the likelihood of finding a compatible donor (4). The presence of DSAs, especially with high mean fluorescence intensity (MFI) or positive crossmatches, is associated with increased risk of antibody-mediated rejection (AMR) and subsequent graft loss (5–7).

In addition to incompatibility, suboptimal HLA matching has emerged as an important determinant of long-term transplant outcomes. Numerous studies have demonstrated that inadequate HLA class II compatibility, particularly at the DQ locus, contributes to the formation of *de novo* DSAs and chronic AMR (8–10). Moreover, a growing body of evidence supports the use of molecular-level matching through eplet-based algorithms to more accurately predict immunologic risk and improve graft survival (11–13).

Kidney paired donation (KPD) programs have revolutionized the field by enabling incompatible donor-recipient pairs to exchange kidneys with other pairs, thus achieving immunologic compatibility through logistical optimization rather than biological matching alone (14, 15). KPD programs have evolved further to incorporate compatible pairs with poor HLA matching, allowing them to access better-matched donors, improve graft longevity, and increase the overall transplant opportunities within the system (16).

Global experiences with KPD vary. The National Kidney Registry (NKR) in the United States and national programs in the UK, Canada, Australia, and the Netherlands have successfully demonstrated that large-scale, multi-institutional KPD networks can efficiently facilitate high-volume exchanges, including chains initiated by non-directed altruistic donors (17) (18–21). However, centralized programs are often constrained by geographic, logistical, and regulatory challenges—particularly in regions with fragmented healthcare systems.

In contrast, single-center KPD programs offer greater agility, control, and faster execution of exchanges. When supported by a robust matching algorithm, clinical expertise, and desensitization protocols, these programs can achieve outcomes comparable to national networks, particularly in high-volume transplant centers.

Our institution, a high-volume kidney transplant center in Saudi Arabia, has implemented a dynamic, multi-strategy KPD program that includes HLA-incompatible (HLAi), ABO-incompatible (ABOi), and compatible pairs with suboptimal immunologic profiles. In 2024 alone, our center performed 135 KPD transplants—the highest known single-center annual volume to date. This study describes the design, evolution, and outcomes of our single-center KPD program, offering a practical framework for other institutions facing similar challenges in donor compatibility and transplant access.

## 2 Methods

### 2.1 Patient selection, matching algorithm, and data sources

We conducted a study of all patients who underwent kidney transplantation via KPD at our center between January and December 2024. The analysis was limited to the 2024 cohort because this year represented the peak annual KPD transplant volume at our center and, to our knowledge, one of the highest single-center yearly volumes reported worldwide. Evaluating this period also allowed assessment of the operational feasibility of executing high-volume exchange activity within a single-center program. The primary objective of this study is to describe the key operational strategies to run an effective KPD program. This Study was conducted in accordance with the principles of the Istanbul Declaration.

Matching of donor–recipient pairs was performed using the commercially available Kidney Match for Life software platform (22).The process began by defining the eligible participant pool and configuring global and patient-specific variables that governed immunologic compatibility, logistical feasibility, and clinical constraints. Upon execution, the system first identified all valid donor–recipient “swaps,” which served as the foundational units for constructing exchange cycles and chains. Two algorithmic approaches were available: an optimization mode and a transparent (exhaustive) mode. The optimization mode generated a single optimal subset of matches in which no donor or recipient appeared in more than one exchange, with default settings configured to maximize the total number of transplants while allowing user-defined weighting parameters to prioritize qualitative factors such as HLA compatibility, sensitization status, and demographic proximity. The transparent mode performed an exhaustive search to display all feasible matching combinations within predefined chain and loop length limits, enabling post-processing filtering and manual selection when required. Additional functionalities included patient-centric matching, which allowed targeted runs for individual candidates, and a domino chain builder for manual chain construction. Match runs were initiated once the active registry reached a minimum threshold of 50 incompatible donor–recipient pairs, a cutoff determined through internal simulation to ensure productive outcomes. Subsequent runs were triggered by significant pool modifications, including addition or removal of participants, updates to compatibility settings, or revisions of unacceptable antigen profiles in sensitized patients. *Ad hoc* match runs were also performed in cases of clinical urgency, with temporary adjustment of algorithmic parameters—such as permitting ABO-incompatible options—when necessary to broaden the search scope and enhance the likelihood of identifying suitable matches.

Data were collected prospectively from the hospital’s electronic medical records and the organ transplant registry.

Next-generation sequencing (NGS) HLA typing (Gendex NGS engine 3.1) was performed for all HLA loci on all registered donor-recipient pairs. HLA antibodies were analyzed by HLA single antigen assay (One lambda HLA Fusion 4.7). For sensitized patients, the extent of HLA sensitization—reflected by calculated panel reactive antibody (cPRA)—was assessed using the OPTN calculator, applying a MFI threshold of 1000. Prior to transplantation, all candidates underwent T-cell and B-cell IgG flow cytometric crossmatch testing.

For HLA-incompatible candidates with a cPRA greater than 80%, the following locus-specific MFI thresholds were used to guide donor matching:

- 4000 for HLA-A, -B and -DR
- 5000 for HLA-DQB
- 20000 for HLA-C and -DP

### 2.2 Kidney paired donation implemented strategies

Our center has developed the following strategies to execute KPD program.

a) Inclusion of compatible pairs with suboptimal HLA Matching: Compatible donor-recipient pairs with suboptimal HLA matching were included to improve HLA class II matching, reduce class II eplet mismatch load, and achieve better Living Kidney Donor Profile Index (LKDPI) score (23). Additionally, this strategy helps to balance blood type distribution within the donor and recipient pools (enriching donor pool with blood type O donors), thus facilitating better matching for HLA-incompatible (HLAi) and ABO-incompatible (ABOi) candidates.
b) ABO quasi-compatible Matching: ABO quasi-compatible matches (A2 to O, A2 to B, and A2B to B) were permitted for all KPD candidates.
c) Low-Risk HLA-Incompatible Matching: This strategy targeted HLA-incompatible kidney candidates with a cPRA greater than 80%. Low-risk HLA-incompatible matching was defined as the presence of DSAs against the exchanged donor and a positive T-cell and/or B-cell IgG flow cytometric crossmatch, with a channel shift of less than 300 on a 1024 scale. Additionally, the DSA must not be directed against a mismatched paternal antigen in a multiparous woman or against a repeat mismatch from a previous solid organ transplant.
d) ABO-Incompatible Matching: This strategy targeted very highly sensitized HLAi candidates (cPRA >95%). All types of ABOi combinations were permitted, except AB donors to O recipients. Baseline isoagglutinin titers were ≤64 for both IgM and IgG.

As described above, high-frequency match runs were performed with a cutoff pool size of 50 pairs. The pool size did not exceed 110 pairs during any match run. Flexible exchange structures were permitted, including paired exchanges (2-way), 3- to 5-way closed chains, and domino chains. Priority in matching was given to candidates with a cPRA ≥ 95% and to blood group O recipients of non-A2 ABO-incompatible donors. Additionally, exchanges with superior HLA class II matching were prioritized.

### 2.3 Immunosuppressive protocols

All patients received anti-thymocyte globulin (ATG^®^) for induction. An initial dose of 1.5 mg/kg was administered intraoperatively before arterial clamp release. Two additional doses were given on postoperative days 1 and 2. A fourth dose was administered if CD3/lymphocyte counts warranted, with a maximum of four doses.

#### 2.3.1 For flow crossmatch-positive patients

Rituximab (500 mg Intravenous [IV] over 8–10 hours) and IV immunoglobulin (IVIG, 2 g/kg over 24–48 hours) were administered preoperatively.

#### 2.3.2 For ABO-incompatible patients

Rituximab (500 mg IV over 8–10 hours) was administered pre-transplant. Plasmapheresis was performed to reduce ABO titers to <1:8 for both IgG and IgM. Each plasmapheresis session was followed by IVIG at 100 mg/kg.

#### 2.3.3 Maintenance immunosuppression

All patients received triple immunosuppressive therapy consisting of Prednisone, Tacrolimus and Mycophenolate mofetil.

### 2.4 Statistical analysis

Continuous variables were summarized as mean ± standard deviation (SD) and compared across groups using the Kruskal–Wallis test, as appropriate for non-normally distributed data. Categorical variables were presented as frequencies and percentages, with intergroup differences assessed using the Chi-square test or Fisher’s exact test when expected cell counts were low. A two-tailed p-value of < 0.05 was considered indicative of statistical significance. All analyses were performed using R version 4.4.3.

## 3 Results

### 3.1 Patient demographics and baseline characteristics

A total of 135 patients underwent kidney transplantation through our single-center Kidney Paired Donation (KPD) program in 2024, including 121 adults and 14 pediatric recipients (**Table 1**). Among these, 69 patients (51.1%) were HLA-incompatible (HLAi) with their intended donors, 37 patients (27.4%) were ABO-incompatible (ABOi), and 29 patients (21.5%) were compatible pairs.

**Table 1:** Baseline demographic and clinical characteristics of all patients undergoing kidney transplantation through the KPD program in 2024, stratified by compatibility group.

Variable	All Patients	HLA Incompatible	ABO Incompatible	Compatible	p-value
Total. N (%)	135	69 (51.1)	37 (27.4)	29 (21.5)	
Age. mean ± (SD)	42.5 (18.3)	49.9 (14.9)	34.5 (19.5)	35.0(17.4)	<0.001
Females. N (%)	82 (60.7)	54 (78.3)	17 (45.9)	11 (37.9)	<0.001
Causes of ESRD. N (%)					0.17
Unknown	55 (40.7)	36 (52.2)	11 (29.7)	8 (27.6)	
Diabetes	32 (23.7)	17 (24.6)	8 (21.6)	7 (24.1)	
GN	22 (16.3)	8 (11.6)	8 (21.6)	6 (20.7)	
Others	11 (8.1)	3 (4.3)	4 (10.8)	4 (13.8)	
FSGS	9 (6.7)	2 (2.9)	3 (8.1)	4 (13.8)	
Urology	6 (4.4)	3 (4.3)	3 (8.1)	0 (0.0)	
First transplant. N (%)					0.02
1st	118 (87.4)	55 (79.7)	35 (94.6)	28 (96.6)	
2nd+	17 (12.6)	14 (20.3)	2 (5.4)	1 (3.4)	
CPRA. Median (IQR)	57.6 (0.0-94.2)	92.6 (63.7-97.3)	0.0 (0.0-38.0)	0.0 (0.0-12.0)	<0.001
Post-Exchange compatibility status. N (%)					<0.001
ABOI	3 (2.2)	3 (4.3)	0 (0.0)	0 (0.0)	
ABOQC	14 (10.4)	4 (5.8)	7 (18.9)	3 (10.3)	
ABOQC+HLAI	1 (0.7)	1 (1.4)	0 (0.0)	0 (0.0)	
Compatible	94 (69.6)	36 (52.2)	31 (83.8)	27 (93.1)	
HLAI	23 (18.5)	25 (36.2)	0 (0.0)	0 (0.0)	
KDPI mean ± (SD)	-5.8(18.3)	-3.9 (19.1)	-5.3 (18.8)	-11.2 (14.8)	0.29
Class II EpMM mean ± (SD)	22.2 (15.7)	20.1 (15.3)	23.8 (14.8)	25.3 (17.4)	0.19
DQ EpMM (mean ± SD)	10.1 (7.4)	8.6 (7.3)	11.4 (7.1)	12.0 (7.2)	0.02
Outcomes					
Patient died. N (%)	1 (0.7)	1 (1.4)	0 (0.0)	0 (0.0)	>0.9
Graft Failed. N (%)	1 (0.7)	1 (1.4)	0 (0.0)	0 (0.0)	>0.9
ACR. N (%)	9 (6.7)	5 (7.2)	3 (8.1)	1 (3.4)	0.72
AMR. N (%)	1 (0.7)	1 (1.4)	0 (0.0)	0 (0.0)	0.62
CAAMR. N (%)	2 (1.5)	1 (1.4)	1 (2.7)	0 (0.0)	0.67
CATCMR. N (%)	0 (0.0)	0 (0.0)	0 (0.0)	0 (0.0)	NA
*Creatinine. mean ± (SD)	87.2 (31.5)	85.50 (27.4)	88.4 (39.1)	89.7 (30.4)	0.80

ESRD, End Stage Renal Disease; GN, Glomerulonephritis; FSGS, Focal Segmental Glomerulosclerosis; ABOI, ABO Incompatible; ABOQC, ABO Quasi Compatible; HLAI, Human Leukocyte Antigen Incompatible; KDPI, Kidney Donor Profile Index; EpMM, Epilet mismatch; ACR, Acute Cellular Rejection; AMR, Antibody Mediated Rejection; CAAMR, Chronic Active Antibody Mediated Rejection; CATCMR, Chronic Active Antibody T Cell Mediated Rejection, * (µmol/L)..

Females comprised 60.7% of the cohort, with a significantly higher proportion in the HLAi group compared to ABOi and compatible pairs (p < 0.001). The overall mean recipient age was 42.5 years, with HLAi recipients being significantly older than those in the other groups (p < 0.001). Post-exchange, the cohort had a mean Living Kidney Donor Profile Index (LKDPI) of –5.8. The mean class II eplet mismatch was 22, and the mean DQ eplet mismatch was 10. Using a four-antigen matching scheme for HLA class II (DR and DQ), 0 HLA class II mismatches were achieved in 20 patients (15%), 1 mismatch in 28 patients (21%), and 2 mismatches in 66 patients (49%). Notably, 45 patients (33%) achieved 0 HLA-DQ mismatches.

### 3.2 KPD exchange modalities

Of the 135 transplants performed, 126 patients (93.3%) underwent transplantation via 2- to 5-way closed chains, while 9 patients (6.7%) received transplants through domino chains. Among those transplanted through closed chains, 42 patients (33.3%) participated in 2-way exchanges, 45 patients (35.7%) in 3-way exchanges, 24 patients (19.0%) in 4-way exchanges, and 15 patients (11.9%) in 5-way exchanges.

### 3.3 Sensitization and ABO status at time of registration

**Figure 1:**
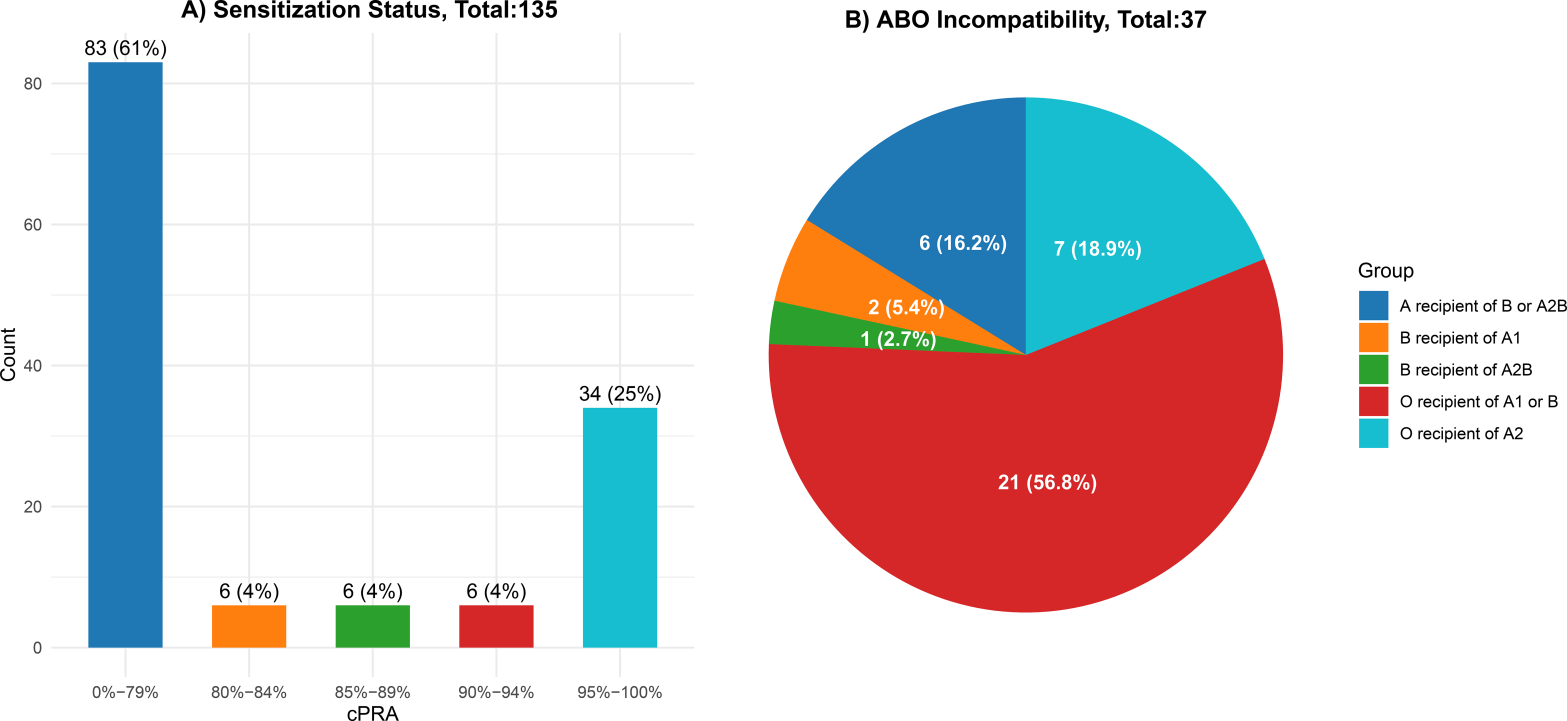
Sensitization and ABO incompatibilities at registration. **(A)** Sensitization Status: Displays the distribution of sensitization levels among a total of 135 participants. The bar graph indicates the percentage of individuals categorized by their calculated Panel Reactive Antibody (cPRA) levels: 0%-79% (61%), 80%-89% (25%), and 90%-100% (14%). **(B)** ABO Incompatibility: Illustrates the breakdown of ABO incompatibility among a total of 37 recipients. The pie chart shows the percentage of recipients for each blood type category: recipients of B or A1 (57%), recipients of A2 (19%), recipients of A2B (16%), and recipients of B (8%).

At registration, 61% of patients had a calculated panel reactive antibody (cPRA) between 0–79%, 12% had cPRA between 80–94%, and 25% were very highly sensitized with cPRA ≥95% (**Figure 1**). Among the 37 ABOi patients, the majority (57%) were blood group O recipients of either A1 or B donors. ABO-incompatibilities at registration are detailed in **Figure 1**.

### 3.4 Compatibility achieved through KPD matching

**Figure 2:**
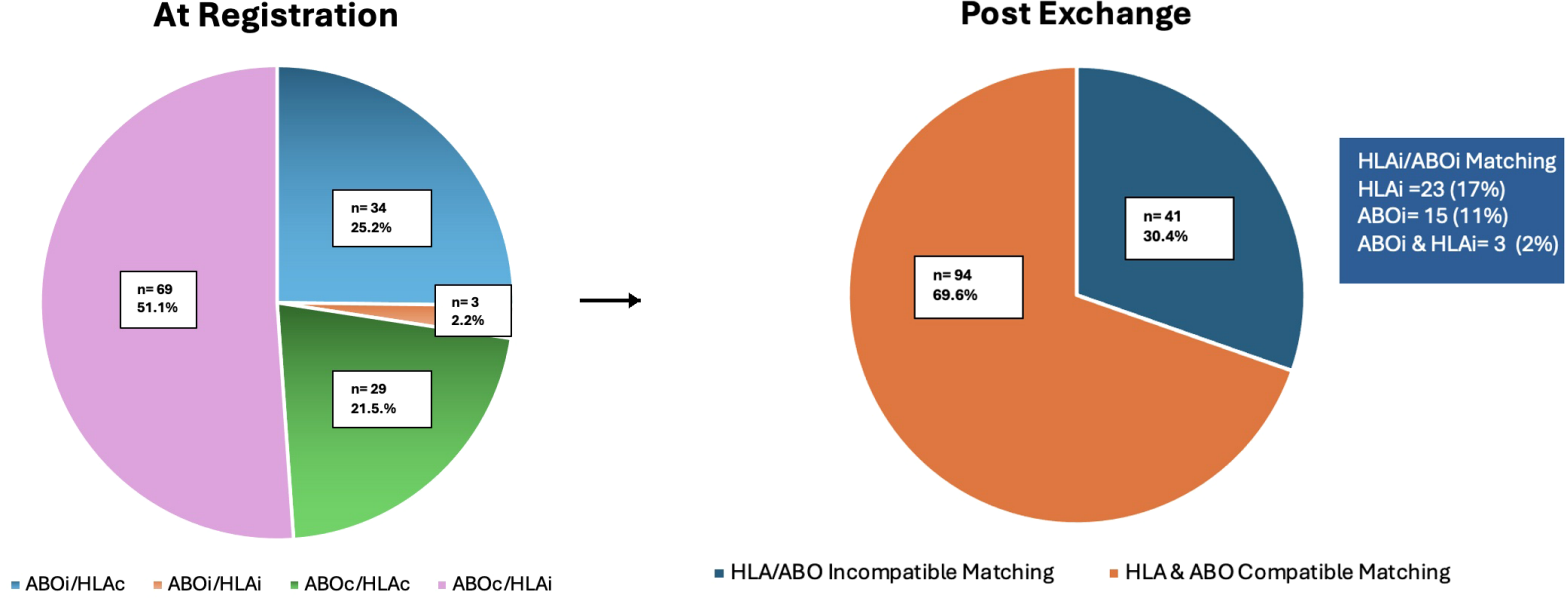
Distribution of Participant Groups across Matching Categories. The left pie chart illustrates the distribution of 135 participants across four groups: ABOi/HLAc (25.2%, n = 34), ABOi/HLAi (2.2%, n = 3), ABOc/HLAc (21.5%, n = 29) and ABOc/HLAi (51.1%, n=69). The right pie chart represents the post exchange status: 94 (69.6%) received kidneys from the HLA and ABO compatible donors while 41 (30.4%) participants received kidneys by HLA/ABO incompatible matching. The colored text box shows the distribution of HLA/ABO incompatible matching.

Following execution of the optimized matching algorithm, 94 patients (70%) achieved full ABO and HLA compatibility with the exchanged donor. The remaining 41 patients (30%) were transplanted under low-risk HLA-incompatible or ABO-incompatible conditions. **Figure 2** and **Table 2** illustrate the distribution of compatibility types post-matching.

**Table 2:** Data derived from exchanges through ABO incompatible or Low Risk HLA Incompatible Matching (Total=41).

ABO Incompatible Matching (N = 18)					
Incompatibility	A2 → O	B → A	B → O	A1B → B	A2B → B
Total Number	12	1	1	1	3
Low Risk HLA Incompatible Matching (N = 23)					
FCXM	Total	Channel Shift	DSA class I	DSA class II	DSA class I & II
B +	11	97-283	0	11	0
T+ & B+	12	T:64-216 B:76-246	8	1	3

FCXM, Flow Cytometric Crossmatching; DSA, Donor-Specific Antibodies.

### 3.5 Post-transplant outcomes

During the follow up (ranging from 3 to 15 months), serum creatinine levels were comparable across groups, with an overall mean of 87.2 µmol/L. One patient died and one experienced graft failure—both from the HLAi group (0.7%). Acute cellular rejection occurred in 9 patients (6.7%), with no significant differences among groups (p = 0.72). Antibody-mediated rejection occurred in one ABOi recipient, while chronic AMR was diagnosed in two patients—one each from the HLAi and ABOi groups (1.5%). No cases of chronic T cell–mediated rejection were reported.

## 4 Discussion

This study highlights the feasibility, flexibility, and effectiveness of a high-volume, single-center kidney paired donation (KPD) program in addressing immunologic barriers to transplantation, especially in regions without access to large-scale national registries. Our center’s ability to facilitate 135 KPD transplants in one year—including HLA-incompatible (HLAi), ABO-incompatible (ABOi), and compatible pairs—demonstrates the value of a pragmatic, inclusive strategy that balances immunologic risk with clinical opportunity.

To our knowledge, this represents the highest single-center volume of kidney paired donation transplants reported globally within a calendar year. While national and regional registries such as the National Kidney Registry (NKR) in the United States, United Kingdom Living Kidney Sharing Scheme, or the Canadian Living Donor Paired Exchange Program facilitate hundreds of KPD transplants annually across multiple centers, the number of KPD procedures performed at any one institution typically ranges from several dozen to less than 100 per year. The NKR reported that 116 KPD transplants were performed in Penn Medical Institute in the past 12 months (24).On the other hand a total of 70 KPD transplants were carried out in Canada (25). In the whole UK, 179 kidney transplants were performed through KPD (17). In contrast, our program facilitated 135 transplants in 2024 alone, underscoring the operational capacity, algorithmic optimization, and institutional commitment required to achieve such high-volume performance. This volume reflects not only clinical throughput but also the success of our inclusive matching strategy and streamlined coordination within a single-center framework.

As one of the largest transplant centers in Saudi Arabia, our center performed 511 kidney transplants in 2024, of which 453 were from living donors. KPD accounted for nearly 30% of our living donor transplants. Through targeted strategies — notably the inclusion of compatible pairs into the KPD program — we successfully maintained a balanced ABO blood group distribution between donors and recipients. Individuals with blood group O comprise 55% of recipients and 48% of donors in our pool, encompassing matched, unmatched, and transplanted pairs. This achievement addresses a significant concern previously raised in the literature, where the unbalanced representation of blood group O donors in the donor pool was associated with declining match rates (26, 27). By proactively managing ABO representation, we have thus far been able to preserve match rates and optimize transplant opportunities for patients across all blood groups.

Match rates in KPD programs are influenced by multiple factors. A study by the NKR demonstrated that both cPRA levels and blood group type significantly impact the likelihood of matching (28).Specifically, potential recipients with cPRA >80% or those with blood group O were less likely to find suitable matches within the KPD pool. Since the inception of our KPD program in 2016, we have successfully transplanted 694 patients through different strategies (unpublished data). Our data show a match rate of 73% for ABOi patients and 79% for patients with cPRA >80%. Importantly, among very highly sensitized patients with cPRA between 95–100%, the match rate remained favorable at 74%, highlighting the effectiveness of our program in addressing the challenges associated with difficult to match recipients. However, 118 patients remain unmatched. Of these, 84 (71%) are potential recipients with blood group O, whereas only 29% of the potential donor pool has blood group O. In addition, 64% of patients in the unmatched group have a cPRA >80%.

Not all KPD programs include compatible pairs in their pool. Several reasons have been cited for this, including concerns about creating imbalanced exchanges, fears of weakening the emotional bond through indirect donation, and logistical challenges. However, with appropriate counseling, many compatible pairs may choose to participate in KPD programs (29). The key question is how to provide benefit to compatible patients. They should ideally achieve better HLA matching, improved Class II matching, lower LKDPI scores, and reduced class II eplet mismatch loads. Better HLA matching has been associated with improved graft survival (30). Additionally, a higher eplet mismatch load, particularly in Class II, has been linked to poorer graft outcomes (31, 32). We prioritize securing better HLA matching, lower LKDPI scores, and reduced class II eplet mismatches for compatible pairs compared to their original donors. This approach can also be adopted by newly developing KPD programs or used to strengthen single-center KPD initiatives.

Another strategy in our program involves converting high-risk HLA incompatibility to low-risk incompatibility and then offering desensitization using rituximab and IVIG. Prior studies have shown that desensitized patients have significantly better survival outcomes compared to those who remain on dialysis (33, 34). Almost 25% of our cohort consisted of very highly sensitized patients (cPRA >95%), whom we were able to transplant through a combination of targeted strategies. This approach enabled us to expand transplant opportunities for patients who would otherwise have had limited or no access to transplantation.

A major strength of our study is the successful implementation of a high-volume, single-center KPD model that accommodates a diverse array of clinical scenarios. Unlike national programs constrained by geography and coordination complexity, our program offered agility and continuity of care, particularly valuable in regions with limited transplant infrastructure for KPD. Furthermore, our structured inclusion of compatible pairs not only enhanced individual outcomes but also increased overall exchange opportunities, amplifying the network effect within our center.

Nonetheless, several limitations merit consideration. First, this was a single-center study with a relatively short follow-up period, which limits the ability to assess long-term outcomes such as graft survival, chronic rejection, and the development of DSAs. While early post-transplant outcomes are encouraging, extended follow-up is essential to confirm durability. However, the favorable immunologic profiles of our cohort—reflected in improved HLA class II matching, reduced class II eplet mismatch loads, and low LKDPI scores—suggest a strong potential for superior long-term outcomes. Second, the generalizability of our findings may be limited, particularly for lower-volume centers or those without established desensitization protocols and strong immunologic infrastructure.

In conclusion, our experience demonstrates the operational feasibility of a high-volume, single-center kidney paired donation program as a strategy to address immunologic incompatibilities in kidney transplantation. Through the structured inclusion of HLA-incompatible pairs, ABO-incompatible pairs, and selected compatible pairs seeking improved matching, together with the use of desensitization protocols when required, such programs can expand transplant opportunities for patients with elevated immunologic risk. While longer follow-up and broader comparative analyses are warranted, our findings suggest that this model may provide a practical framework for other high-capacity centers seeking to increase transplant activity and manage complex donor–recipient incompatibilities.

## Data Availability

The original contributions presented in the study are included in the article/supplementary material. Further inquiries can be directed to the corresponding author/s.
